# DNA sensors are expressed in astrocytes and microglia *in vitro* and are upregulated during gliosis in neurodegenerative disease

**DOI:** 10.1002/glia.22786

**Published:** 2015-01-27

**Authors:** Donal J. Cox, Robert H. Field, David G. Williams, Marcin Baran, Andrew G. Bowie, Colm Cunningham, Aisling Dunne

**Affiliations:** ^1^ Molecular Immunology Group, School, of Biochemistry and Immunology and Immunology Research CentreTrinity College DublinIreland; ^2^ Neurodegeneration and CNS Inflammation Group, School of Biochemistry and Immunology and Immunology Research CentreTrinity College DublinIreland; ^3^ Viral Evasion Group, School of Biochemistry and Immunology and Immunology Research CentreTrinity College DublinIreland

**Keywords:** nucleic acid sensing, microglia, astrocytes, Type I interferon, neurodegeneration

## Abstract

The detection of nucleic acids by the innate immune system is an essential host response during viral infection. In recent years, a number of immune sensors capable of recognizing cytosolic DNA have been identified and include the PYHIN family members AIM2, IFI16, and p204 as well as the enzyme, cGAS. Activation of these receptors leads to the induction of antiviral genes including Type‐1 interferons and chemokines such as CCL5. We have carried out extensive expression profiling of these DNA sensors and other members of the PYHIN family in highly purified primary astrocytes and microglia and have demonstrated that both cell types express the majority of these proteins at the mRNA level. In microglia, several family members are highly upregulated in response to IFN‐β treatment while both cell types induce robust proinflammatory and antiviral cytokine production (e.g., IL‐6, CCL5, IFN‐β) in the presence of immune stimulatory DNA and RNA. The production of IL‐6 is partially dependent on the interferon receptor as is IFN‐β itself. Furthermore, we have found that p204 and AIM2 are upregulated in a Type I IFN dependent fashion *in vivo*, in a murine model of chronic neurodegeneration. Given the propensity of inflammatory responses to cause neuronal damage, increased expression and activation of these receptors, not only during viral infection but also during sterile inflammatory responses, has the potential to exacerbate existing neuroinflammation leading to further damage and impaired neurogenesis. GLIA 2015;63:812–825

## Introduction

The recognition of nucleic acids is an essential strategy employed by host immune cells to combat viral pathogens (Unterholzner, 2013). The sensors and pathways involved in RNA/DNA detection have only recently been discovered and have expanded over the last number of years. RNA released into the cytosol is recognised by MDA‐5 and RIG‐I while cytosolic DNA is detected by members of the PYHIN family (also referred to as AIM2 (Absent in Melanoma‐2)‐like receptors, ALRs) in addition to DAI (DNA‐dependent activator of interferon‐regulatory factors) and the enzyme cGAS (cyclic GMP‐AMP synthase). The PYHIN family of proteins are so called as they contain an N‐terminal PYRIN domain and a C‐terminal HIN (haemopoietic expression, interferon‐inducibility, nuclear localization) domain (Schattgen and Fitzgerald, 2011). Of these, AIM2, p204 (also known as Ifi204) and IFI16 have been shown to bind DNA directly via their HIN domains culminating in the production of antiviral and proinflammatory cytokines. Moreover, some members of the PYHIN family (e.g., AIM2 and IFI16) can form inflammasome complexes, resulting in interleukin‐1β (IL‐1β) processing by caspase‐1 (Burckstummer et al., 2009; Fernandes‐Alnemri et al., 2009; Hornung et al., 2009; Roberts et al., 2009). In the case of IFI16, this occurs following recognition of Kaposi's sarcoma‐associated herpesvirus (KSHV) genomic DNA in the cell nucleus (Kerur et al., 2011).

Five PYHIN family members have been identified in humans; IFIX, IFI16, MNDA, AIM2, and POP3 (Keating et al., 2011; Khare et al., 2014) while 13 have been found in mice (Cridland et al., 2012). AIM2 is the only protein with a direct murine homolog, whereas murine p204 is considered a functional ortholog of IFI16. On binding dsDNA, IFI16/p204 signals by directly engaging the ER bound protein, STING (Stimulator of Interferon Genes). STING in turn activates the TBK1/DDX3 complex to induce IFN‐β via phosphorylation of the transcription factor, IRF‐3 (Unterholzner et al., 2010). cGAS, the newest member of the DNA sensing family, catalyses the conversion of the ATP and GTP to the second messenger, cyclic GMP‐AMP (cGAMP). It was recently demonstrated that cGAS binds cytosolic DNA and produces cGAMP, which interacts with STING leading also to the activation of IRF3 and production of IFN‐β (Sun et al., 2013; Wu et al., 2013). cGAS deficient mice exhibited a profoundly lower induction of Type I IFNs, higher viral titers in the brain and higher mortality rates when compared with wild‐type mice in a HSV‐1 infection model (Li et al., 2013). Furthermore, cGAS has been implicated in the control of West Nile Virus (WNV) suggesting a central role for this protein in combating both RNA and DNA viral infection (Schoggins et al., 2014).

In addition to double stranded DNA viruses, a number of RNA viruses can target the CNS including rabies virus (VSV) and Human Immunodeficiency Virus (HIV). Symptoms range from mild to severe and include fever, headache, impaired consciousness, seizure, dementia, or even death (Carty et al., 2014). The UK reports ∼700 cases of encephalitis each year, of which 7% are fatal; however, this number is thought to be much higher due to unreported instances. The US spends ∼$650 million a year in hospital related costs for patients suffering from encephalitis alone (Granerod et al., 2010), hence a detailed understanding of the molecular events governing recognition of these pathogens is required to develop effective treatment regimes. There is an emerging, albeit controversial, relationship between certain viruses and neurodegenerative conditions such as Alzheimer's disease and Multiple Sclerosis (Zhou et al., 2013). Independent of viral invasion, there is now also evidence for Type I interferon responses in chronic neurodegenerative diseases including prion disease and amyotrophic lateral sclerosis (Field et al., 2010; Wang et al., 2011). Furthermore, DNA released from damaged or dying cells is immunogenic and increases in cell‐free DNA have been observed in the serum and CSF of patients suffering from a number of conditions including epilepsy, stroke, and traumatic brain injury (Chiu et al., 2005; de Rivero Vaccari et al., 2014; Liimatainen et al., 2013; Tsai et al., 2011). Hence, DNA is now considered a ‘damage’‐associated molecular pattern (DAMP) as well as a pathogen associated molecular pattern (PAMP) and it has been postulated that DNA sensors can detect aberrantly localized DNA and possibly DNA damage within the nucleus itself. A notable example may be IFI16, which can translocate between the nucleus and cytosol (Kerur et al., 2011).

While it has been demonstrated that microglia and astrocytes can engage responses to RNA via TLR3 (Costello and Lynch, 2013; Ribes et al., 2010), RIG‐I and MDA‐5 (De Miranda et al., 2009; Furr et al., 2008, 2010), the contribution of DNA sensors to nucleic acid recognition has not been examined in any great detail in neuroimmune cells. Furthermore, the contribution of individual cell types, in particular astrocytes, has been confounded by difficulties in obtaining pure populations of these cells devoid of contaminating microglia (Holm et al., 2012; Saura, 2007). Finally, most studies reported to date have used synthetic poly (dA:dT) as a DNA mimetic; however, this is known to be reverse transcribed to RNA by RNA pol III and is capable of activating the RNA sensors, RIG‐I and MDA5 (Ablasser et al., 2009; Chiu et al., 2009). We have addressed some of these methodological issues and have now carried out extensive expression profiling of the newly described DNA sensors and additional PYHIN family members in purified astrocytes and microglia and have found that both cell types have the capacity to induce a strong antiviral and pro‐inflammatory response in the presence of immune stimulatory DNA and RNA. Silencing of the key sensors, cGAS, and p204, results in significantly impaired IFN‐β production. Furthermore, we have found that key sensors are upregulated in brain tissue in a murine model of chronic neurodegeneration.

## Materials and Methods

### Animals

C57BL/6 and IFNAR^−/−^ mice were bred and housed under specific pathogen free conditions and procedures were carried out in accordance with regulations and guidelines of the Trinity College Dublin Ethics Committee, the Irish Medicines Board and the Department of Health.

### Primary Mixed Glial and Microglial Cultures

Primary mixed glia cultures were prepared from whole brains of <1‐day‐old (P1) C57BL/6 postnatal mice (BioResources Unit, Trinity College, Dublin, Ireland). Brains were excised, chopped, and placed in cDMEM containing 10% fetal bovine serum, penicillin/streptomycin (10 μg/ml), and l‐glutamine (2 m*M*). Tissue was triturated, the suspension was filtered through a sterile mesh filter (40 μm) and centrifuged (2,000 rpm, 5 min, 20°C). After 24 h, media was replaced with cDMEM containing granulocyte macrophage‐colony stimulating factor (GM‐CSF; 10 ng/mL) and macrophage‐colony stimulating factor (M‐CSF; 20 ng/mL), (R&D Systems, Minneapolis, MN) and cells were grown at 37°C in a 5% CO_2_ humidified environment for 12–14 days, with medium replaced every 3–4 days. Nonadherent microglial cells were isolated by shaking (100 rpm, 2 h at room temperature), tapping and centrifuging (2,000 rpm, 5 min, 20°C). Microglia were plated onto poly‐l‐lysine (50 μg/mL) coated coverslips at 2 × 10^5^ cells/mL. Remaining mixed glial cells were then removed by trypsin‐ethylenediaminetetraacetic acid (EDTA) digestion for 5 min, counted and plated at a concentration of 2.5 × 10^5^ cells/mL.

### Astrocyte Cultures

Primary mixed glial cultures were prepared from whole brains as described above. Tissue was triturated, the suspension was filtered through a sterile mesh filter (40 μm) and centrifuged (2,000 rpm, 5 min, 20°C). After 24 h, media was replaced with cDMEM and every 3 days thereafter. Mixed glia were removed by trypsin‐EDTA digestion for 5 min, cells were centrifuged (2,000 rpm, 5 min) and the pellet was resuspended in magnetic activated cell sorting (MACS) buffer. Astrocyte cultures were depleted of microglia using MACS CD11b beads and depletion columns (Miltenyi Biotech, UK) as per manufacturer's protocol. Resulting astrocytes were resuspended in cDMEM and plated at either 2 × 10^5^ cells/mL for RT‐PCR analysis or 2.5 × 10^5^ cells/mL for cytokine analysis.

### Flow Cytometry

Microglia or astrocyte cultures were washed in sterile PBS, before scraping and gentle dissociation by pipetting. Cells were resuspended in FACS buffer (1% BSA/PBS) containing Fc Block (1 μg/mL). Cells were stained for CD11b‐APCVio770 and GLAST‐PE (Miltenyi Biotech, UK) for 30 min at 4°C. Cells were washed three times and resuspended in FACS buffer. Flow cytometeric data was collected using a FACSCanto II (BD Biosciences) cell analyzer. Microglia were identified by expression of CD11b and astrocytes by GLAST expression. Flow cytometeric analysis was carried out using FLOWJO version 7.6.5.

### Cell Treatments and Transfection

For cytokine and chemokine analysis primary murine mixed glia, microglia, and astrocytes were transfected with poly (dA:dT) (1 μg/mL), low molecular weight poly (I:C) (1 μg/mL) (Invivogen, Toulouse, France) or Vaccinia virus 70 mer (1 μg/mL) synthesized by MWG Biotech as previously described (Unterholzner et al., 2010) using lipofectamine 2000 (Invitrogen). Supernatants were harvested after either 6 or 24 h. Control cultures were treated with lipofectamine 2000 in the absence of nucleic acids. Transfection efficiency of both astrocytes and microglia was determined by flow cytometry using FITC‐labeled Vaccinia virus 70 mer (1 μg/mL). For gene expression studies both microglia and astrocytes were plated at 2 × 10^5^ cells/mL. Cultures were left untreated or treated with 500 (IU/mL) of IFN‐β for 24 h and RNA extracted.

### siRNA Gene Silencing

Primary microglia and astrocytes were transfected with SMARTpool siRNA (200 n*M*, Dharmacon) targeting either cGAS or p204. After 6 h the medium was changed and replaced with 50% DMEM and 50% Optimem containing 20% FCS and l‐glutamine. Cells were then transfected for 6 h with Vv70mer as previously described at either 48 h (for astrocytes) or 72 h (for microglia) post siRNA treatment. Knockdown of the respective genes was assessed by quantitative PCR.

### Elisa

Cytokine and chemokine levels of IL‐6, TNF‐α, CCL5, CCL3, and CXCL2 were quantified from supernatants using R&D DuoSet ELISA kits (R&D Systems, Minneapolis, MN) as per the manufacturer's protocol. For quantification of IFN‐β, 96 well high binding ELISA plates were coated with monoclonal rat anti‐mouse IFN‐β capture antibody (Santa Cruz) in carbonate buffer (1:1,000; 50 µL/well) overnight at 4°C. Plates were washed 5 times with PBS/Tween (0.05%) and blocked in 200 µL 10% FBS/PBS for 2 h at 37°C. Samples and standards (PBLinterferonsource) were loaded (50 µL/well) and incubated at 4°C overnight. Plates were washed 5 times with PBS/Tween (0.05%) and incubated with polyclonal rabbit antimouse IFN‐β detection antibody (50 µL/well) (PBLinterferonsource) overnight at 4°C. Anti‐rabbit‐peroxidase (1:2,000; 50 µL/well) was added and left for 2 h at room temperature. ELISAs were developed in Tetramethylbenzidine (TMB) (50 µL/well) and stopped with 1*M* H_2_SO_4_ (25 µL/well)_._ Optical densities were determined at 450 nm using a microplate reader.

### Quantitative Real‐Time PCR

RNA was extracted using the High pure RNA Isolation Kit (Roche) and cDNA synthesized using GoScript Reverse Transcriptase kit (Promega). Quantitative real time PCR was carried out using iTaq™ Universal SYBR® Green mastermix (Biorad) on a Biorad CFX96 Real‐Time System. mRNA levels were quantified using the primers listed in Table 1. mRNA expression levels were normalized to β‐actin mRNA levels.

**Table 1:** Real‐Time PCR Primers Used in This Study

Gene	Primers
P204	F: 5′‐GTGTGTGGAGAACACAGTTTCATCAAGATATC‐3′
	R: 5′‐GGTTCTGTTACTTTCAGCACCATCACTTG‐3′
Ifi203	F: 5′‐GTGCCAAAACCCGGGAACAAGATATC‐3′
	R: 5′‐CAGTTCCTCTCCTTGGTGCCTTAATTC‐3′
Ifi207	F: 5′‐CCCAGCAGTTCCTCAAACAAGAAACAG‐3′
	R: 5′‐GCTGGGAAGTTGCTGGATGATGAAAATTC‐3′
Ifi202	F: 5′‐CACAGTTTCATCAAGGGAGAAAAGCTACT‐3′
	R: 5′‐CAATGCCACCACTTGTTTGGGACC‐3′
Ifi206	F: 5′‐CCCAGCAGTTACCAAAATTCCCCTC‐3′
	R: 5′‐CTTCAGTCTTGGTTTCTTGGGTGGTG‐3′
PYHIN 1	F: 5′‐GCCAAGAGACAGAGACTGAAAAATGTACC‐3′
	R: 5′‐CATGGAACATCTTCTCTTCTGTCACGTC‐3′
Ifi214	F: 5′‐TCCAGCAGGATTCTGGACCCTCC‐3′
	R: 5′‐AGCTTTGATGACCTTGGCTGGTG‐3′
Ifi208	F: 5′‐GCCACTCAAGGCAAAGATAGGATCTC‐3′
	R: 5′‐CTGGGGATTCTGCATTTCATTGTCCTC‐3′
Ifi213	F: 5′‐GCTCGTGTTGATATACTTAGAAAAGAGATGGAG‐3′
	R: 5′‐GACGGTGTACCTCTGATGAAGCTG‐3′
Ifi205	F: 5′‐GCCCAGAAAAGGAAAGGTATGAGTGAAG‐3′
	R: 5′‐CTGATCTGCTTTCCCAGATGCCTTGATC‐3′
MNDAL	F: 5′‐CCACCAACATCACCCAGCAGTTC‐3′
	R: 5′‐GTTCTGGAAGCTGAGCCTGCTCC‐3′
AIM2	F: 5'‐CACCCTCATGGACCTACACTACCG‐3'
	R: 5′‐CCATAGGGGCTGCTCGATCCAC‐3′
cGAS	F: 5'‐ACCGGACAAGCTAAAGAAGGTGCT‐3'
	R: 5'‐GCAGCAGGCGTTCCACAACTTTAT‐3'
β‐Actin	F: 5′‐TCCAGCCTTCCTTCTTGG GT‐3′
	R: 5′‐GCACTGTGTTGGCATAGAGGT‐3′

### In Vivo Experiments: ME7 Model of Chronic Neurodegeneration

Female C57BL/6 and IFNAR^‐/‐^ mice were housed in groups of five and given access to food and water *ad libitum*. Animals were kept in a temperature‐controlled room (21°C) with a 12:12 h light–dark cycle. The mice were anaesthetised intraperitoneally (i.p.) with Avertin (2,2,2‐tribromoethanol) and positioned in a stereotaxic frame. To induce prion disease, 2 small holes were drilled in the skull either side of the midline to allow for bilateral injection of 1 μL of a 10% (w/v) ME7 scrapie‐infected C57BL/6 brain homogenate made in sterile PBS. Injections were made into the dorsal hippocampus (co‐ordinates from bregma: anteroposterior, −2.0 mm; lateral, −1.6 mm; depth, −1.7 mm) using a microsyringe (Hamilton, Reno, Nevada). Control animals were injected with a 10% (w/v) normal brain homogenate (NBH) in PBS, derived from a naive C57BL/6 mouse. poly (I:C) was obtained from Amersham Biosciences (Little Chalfont, Buckinghamshire, UK). It was prepared for injection by resuspending in sterile saline, heating to 50°C at a concentration of 2 mg/mL to ensure complete solubilisation and then allowing to cool naturally to room temperature to ensure proper annealing of double‐stranded RNA. poly (I:C) was stored at −20°C until use. Experimental groups at 18 weeks postinoculation with ME7 or NBH were challenged intraperitoneally (i.p.) with either poly (I:C) (12 mg/kg) or sterile saline to examine DNA sensor expression. Animals were sacrificed and perfused at 4 h post poly (I:C) injection. Brains were rapidly removed and the area of major pathology (hippocampal and thalamus) was dissected out, snap frozen on liquid nitrogen and stored at −80°C until further use. All procedures were performed in accordance with Republic of Ireland Department of Health & Children license.

### Statistical Analysis

Statistical analysis was performed using Graphpad Prism 5 software. The means for three or more groups were compared by one‐way ANOVA. Where significant differences were found, the Tukey–Kramer multiple comparisons test was used to identify statistical differences between individual groups. For the means of two groups an unpaired Student's *t*‐test was used to identify statistical significance. Group differences were analyzed by two‐way ANOVA with multiple comparisons followed by Bonferroni *post test* comparisons.

## Results

### Nucleic Acids Drive Cytokine and Chemokine Production in Primary Mixed Glia

**Figure 1:**
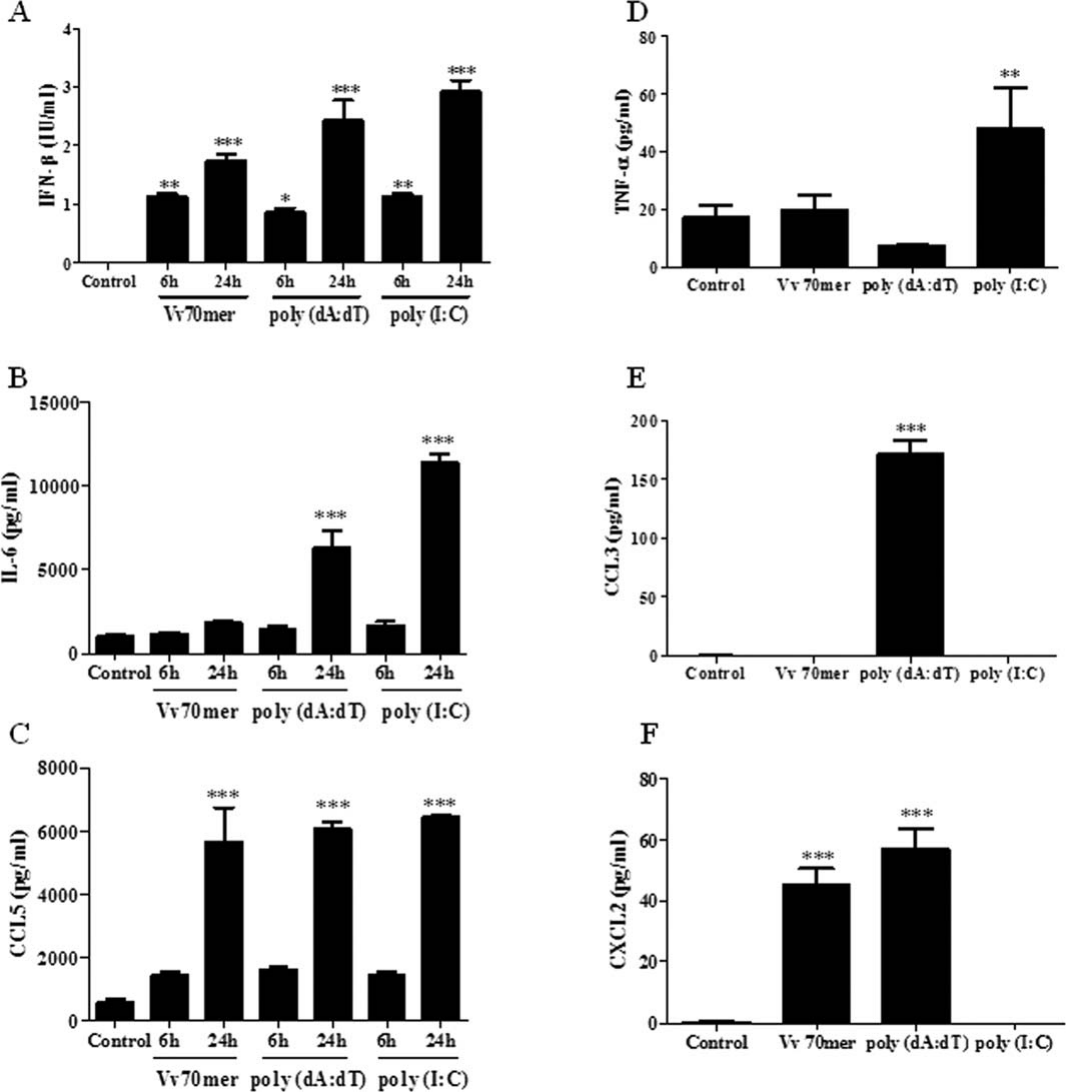
Nucleic acids induce pro‐inflammatory cytokine and chemokine production in primary murine mixed glia. Primary murine mixed glial cultures were transfected with Vv70mer (1 μg/mL), poly (dA:dT) (1 μg/mL) or poly (I:C) (5 μg/mL) for 6 or 24 h (**A–C**) or for 24 h (**D–F**). Supernatants were harvested and IFN‐β (A), IL‐6 (B), CCL5 (C), TNF‐α (D), CCL3 (E), and CXCL2 (F) production was quantified by ELISA. Results shown are means ± SD for triplicate cultures and are representative of three independent experiments.*P ≤ 0.05, **P ≤ 0.01, ***P ≤ 0.001, compared with control.

It has previously been demonstrated that microglia and astrocytes respond to cytosolic RNA (Furr et al., 2008). Preliminary experiments were performed in order to determine if glia respond to cytosolic DNA. The sensors in question will not respond to exogenously added DNA therefore, mixed glial cultures were transfected with a vaccinia virus derived 70 mer (Vv70mer; 1 μg/mL), which cannot be reverse transcribed to RNA or the synthetic DNA mimetic poly (dA:dT) (1 μg/mL). The synthetic RNA mimetic, low molecular weight poly (I:C) (5 μg/mL), was included for comparative purposes. After 6 and 24 h, supernatants were harvested and IFN‐β production was measured by ELISA. All three ligands drove robust IFN‐β production with poly (I:C) giving the strongest response (Fig. 1A). Mixed glia were cultured in the presence of M‐CSF and GM‐CSF in order to enhance microglia cell numbers. Importantly, there was no significant difference in IFNβ production in cells that were cultured in the absence of growth factors (data not shown). IL‐6 production was observed after 24 h in response to both poly (dA:dT) and poly (I:C) (Fig. 1B) while the chemokine CCL5 was induced at high levels in response to all three ligands (Fig. 1C). Like IL‐6, TNF‐α production is under the control of NF‐κB; however, we did not detect this cytokine in samples at either 6 or 24 h in response to DNA. Minimal levels of the cytokine were produced in response to poly (I:C) after 24 h stimulation (Fig. 1D). We also measured the production of CCL3 and CXCL2 as both of these chemokines have been shown to play a role in host defence during CNS infection (Hosking et al., 2009; Trifilo et al., 2003). Cytosolic DNA induced low levels of CCL3 and CXCL2 in comparison to IL‐6 and CCL5. Notably, poly (I:C) did not induce production of either CCL3 or CXCL2 (Fig. 1E,F).

### Constitutive and Inducible Expression of DNA Sensors in Primary Murine Microglia and Astrocytes

**Figure 2:**
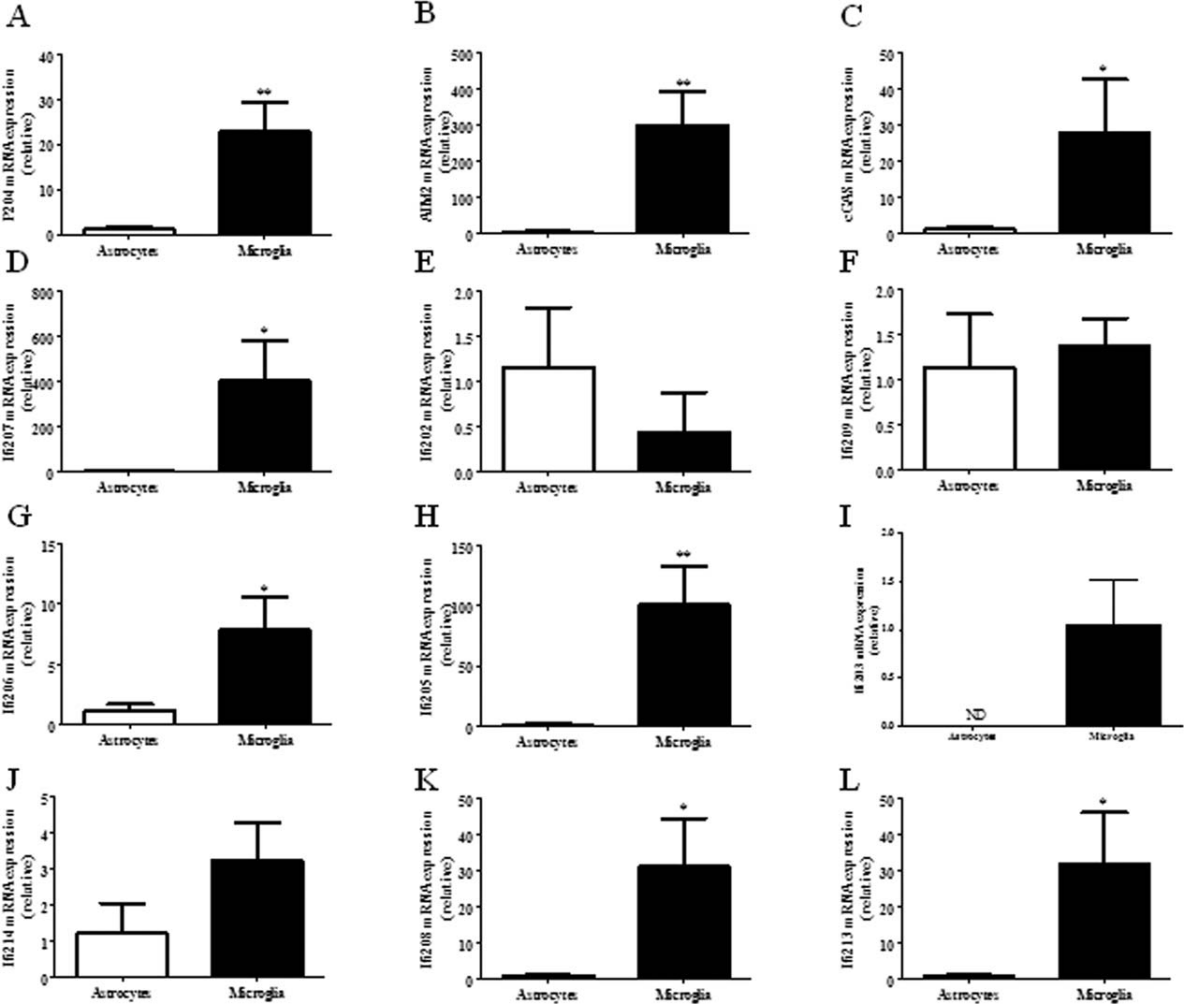
Baseline expression of DNA sensors and other PYHINs in primary murine microglia and astrocytes. RNA was extracted from primary murine microglia and astrocytes and levels of p204 (**A**), AIM2 (**B**), cGAS (**C**), Ifi207 (**D**), Ifi202 (**E**), Ifi209 (**F**), Ifi206 (**G**), Ifi205 (**H**), Ifi203 (**I**), Ifi214 (**J**), Ifi208 (**K**), and Ifi213 (**L**) were analyzed by quantitative PCR. Results shown are means ± SD for triplicate cultures. *P ≤ 0.05, **P ≤ 0.01.

Having shown that glia respond to cytosolic DNA we next examined the basal expression of the known DNA sensors and other members of the PYHIN family in purified astrocyte and microglia populations. We used microglial‐depleting MACS CD11b beads and depletion columns in order to obtain purified astrocyte cultures. Antibodies are not currently available for the majority of these proteins, therefore quantitative real‐time PCR was conducted. For comparative purposes, expression of the genes in astrocytes was standardised and microglial expression was examined relative to them. All PYHINs examined in addition to cGAS were expressed at the mRNA level in both cell types, with the exception of Ifi203 which was not expressed in astrocytes (Fig. 2) and MNDAL, which was not expressed in either cell type (data not shown). In most cases, baseline expression was higher in microglia, with the exception of Ifi202 and Ifi209.This data is summarized in Table 2. Because of the high sequence homology of MNDA to Ifi205, p204, and MNDAL, expression of this gene could not be accurately measured.

**Table 2:** Baseline Expression of DNA Sensors and PYHIN Proteins in Primary Murine Microglia and Astrocytes

Gene	Microglia	Astrocytes
P204	+	+
AIM2	++	+
cGAS	+	+
Ifi207	++	+
Ifi202	+	+
Ifi209	+	+
Ifi206	+	+
Ifi205	++	+
Ifi203	+	—
Ifi214	+	+
Ifi208	+	+
Ifi213	+	+
MNDAL	−	−

++ denotes expression above 100‐fold.

**Figure 3:**
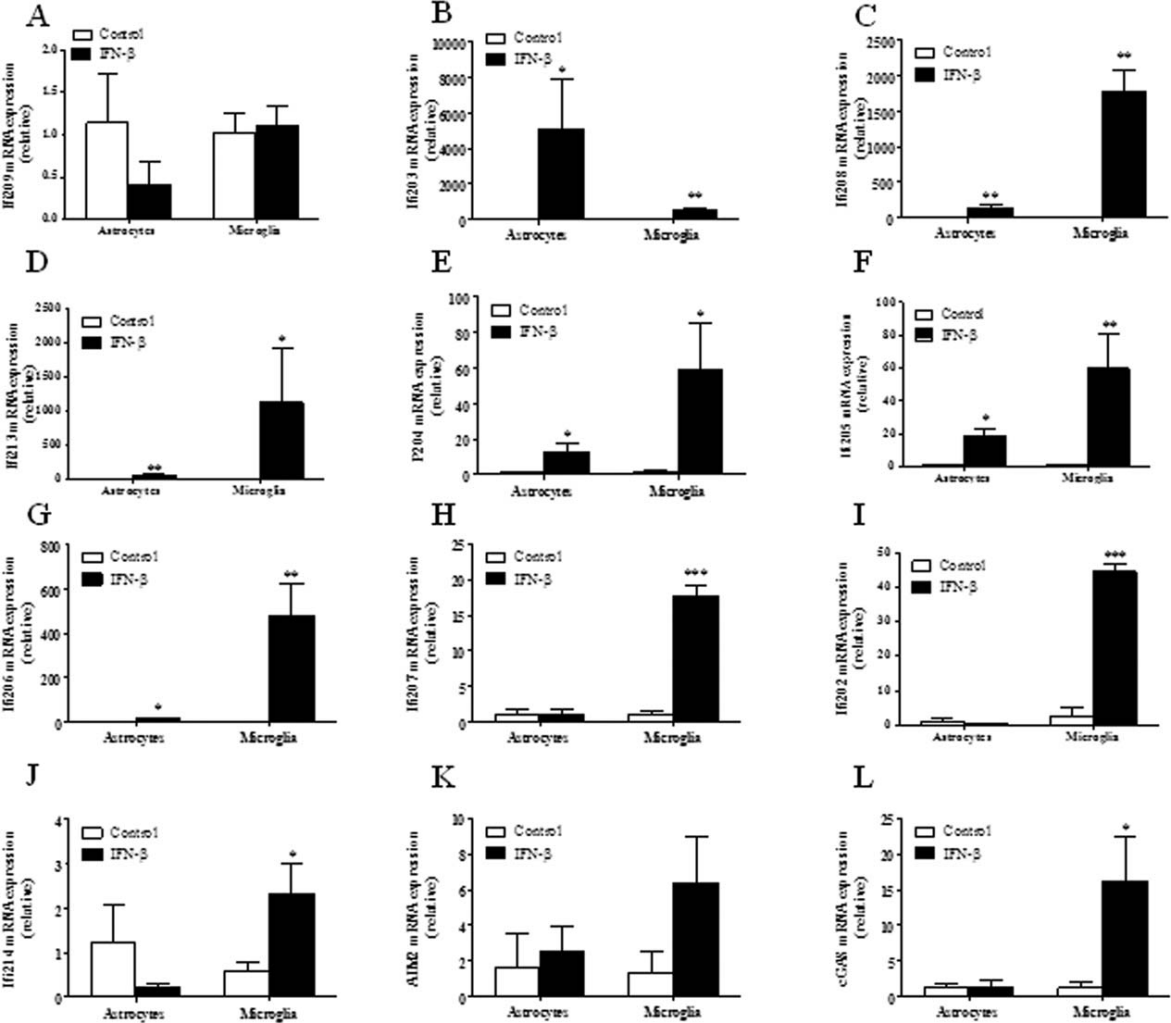
IFN‐β inducible expression of DNA sensors and PYHINs in primary murine microglia and astrocytes. Primary murine microglia and astrocytes were untreated (white bar) or treated with IFN‐β (black bar) (500 IU/mL) for 24 h. RNA was extracted and mRNA levels of Ifi209 (**A**), Ifi203 (**B**), Ifi208 (**C**), Ifi213 (**D**), p204 (**E**), Ifi205 (**F**), Ifi206 (**G**), Ifi207 (**H**), Ifi202 (**I**), Ifi214 (**J**), AIM2 (**K**) and cGAS (**L**) were analyzed by quantitative PCR. Results shown are means ± SD for triplicate cultures. *P ≤ 0.05, **P ≤ 0.01, ***P ≤ 0.001, as compared with respective controls.

Having shown that the majority of DNA sensors and PYHINs are expressed at a basal level in primary microglia and astrocytes, the ability of IFN‐β to enhance their expression was next assessed. Primary murine microglia and astrocytes were treated with IFN‐β for 24 h and gene expression was examined by quantitative real‐time PCR. A robust induction of the PYHINs was observed in microglia (Fig. 3), with the exception of Ifi209 which was not expressed above the basal level (Fig. 3A). IFN‐β –induced gene expression was substantially lower in astrocytes as compared with microglia, with the exception of Ifi203, which was more highly induced in the former (Fig. 3B). The greatest induction was seen with Ifi208 and Ifi213 (Fig. 3C,D); however, as of yet, their biological function remains unknown.

### Astrocytes and Microglia Both Contribute to IFN‐β Production in Response to Nucleic Acids

**Figure 4:**
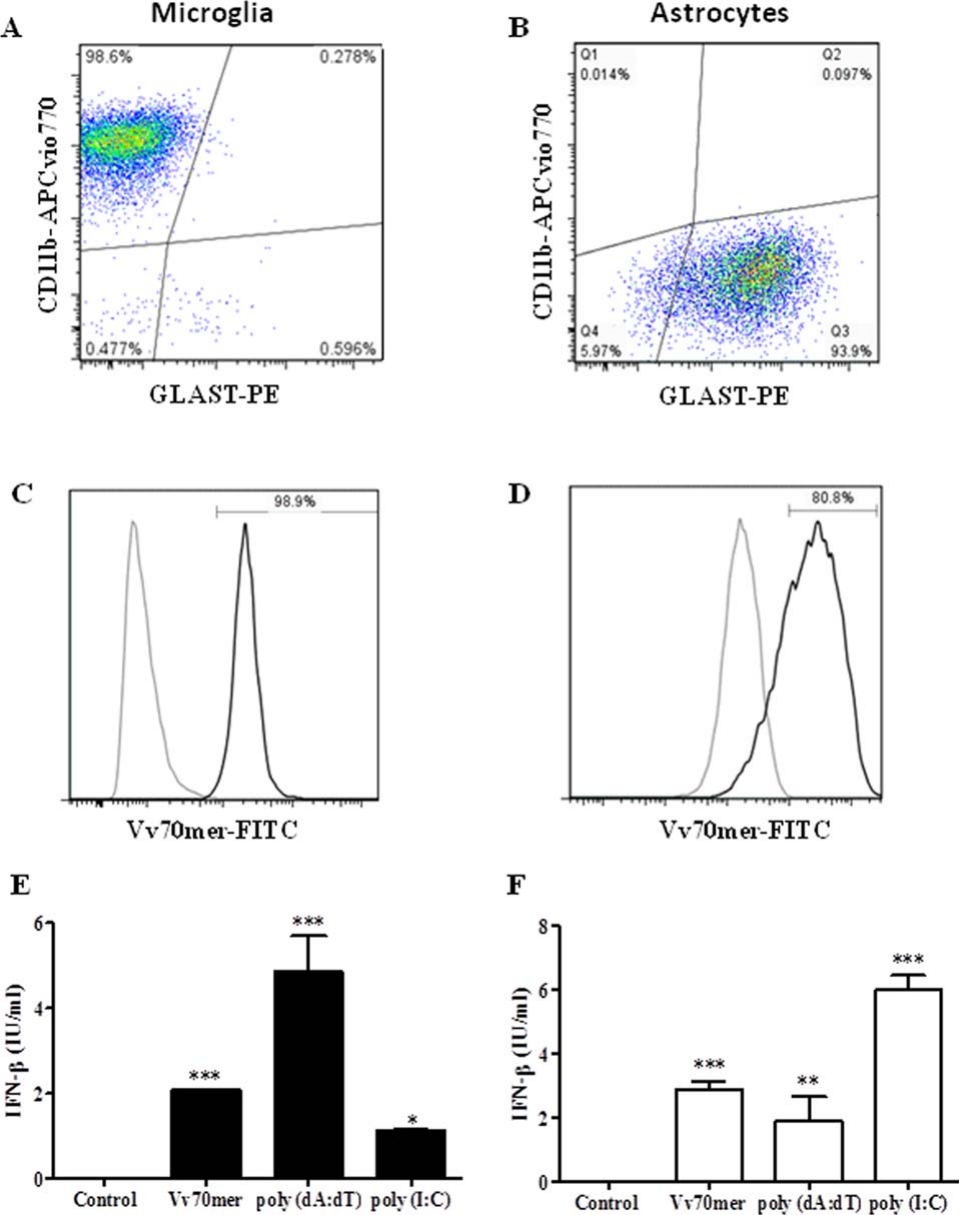
Nucleic acids induce IFN‐β production in primary murine microglia and astrocytes. Primary murine microglia (**A**) and astrocytes (**B**) were transfected with Vv70mer (1 μg/mL), poly (dA:dT) (1 μg/mL) or poly (I:C) (5 μg/mL) for 24 h. Transfection efficiency of microglia (**C**) and astrocytes (**D**) was assessed by flow cytometry after transfection with FITC‐Vv70mer (Untransfected, gray; transfected, black). Supernatants were harvested and IFN‐β production by microglia (**E**) and astrocytes (**F**) was quantified by ELISA. Results shown are means ± SD for triplicate cultures and are representative of three independent experiments. *P ≤ 0.05, **P ≤ 0.01, ***P ≤ 0.001, as compared with control.

We next sought to examine if astrocytes and microglia both contribute to the production of IFN‐β in response to nucleic acid stimulation. Previous studies demonstrating cytokine production by astrocytes in response to innate immune activators have been confounded by residual populations of contaminating microglia in most astrocyte cultures (Saura, 2007). Representative FACS plots illustrating the purified microglia and astrocyte populations used in this study are shown in Fig. 4A,B. The transfection efficiency of both cell types was examined by flow cytometry using FITC‐ labeled Vv70mer DNA. Transfection efficiency was 98% and 80% for microglia and astrocytes, respectively (Fig. 4C,D). These pure cultures were used to demonstrate the induction of IFN‐β at 24 h post‐transfection with the Vv70mer, poly (dA:dT) and poly (I:C). Both Vv70mer and poly (dA:dT) induced strong IFN‐β production, while poly (I:C) elicited a weaker response compared with the DNA stimulations in primary microglia (Fig. 4E). Poly (dA:dT) induced the greatest production of IFN‐β, most likely due to a dual activation of the DNA and RNA sensing pathways. In primary astrocytes, poly (I:C) induced IFN‐β to a greater extent than either the Vv70mer or poly (dA:dT) (Fig. 4F) possibly indicating a greater role for astrocytes in the detection of RNA viruses within the CNS.

### Differential Expression of Proinflammatory Cytokines and Chemokines by Astrocytes and Microglia

**Figure 5:**
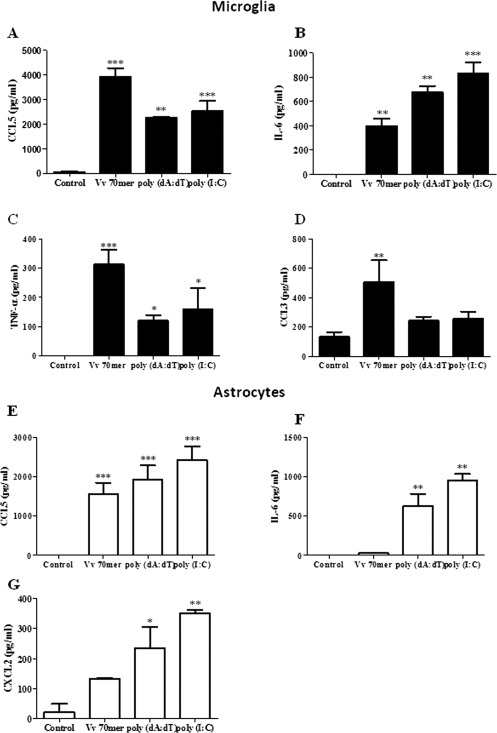
Nucleic acids induce pro‐inflammatory cytokine and chemokine production in primary murine microglia and astrocytes. Primary murine microglia (black bar) and astrocytes (white bar) were transfected with Vv70mer (1 μg/mL), poly (dA:dT) (1 μg/mL) or poly (I:C) (5 μg/mL) for 24 h. Supernatants were harvested and CCL5 (**A**, **E**), IL‐6 (**B**, **F**), TNF‐α (**C**), CCL3 (**D**), and CXCL2 (**G**) production was quantified by ELISA. Results shown are means ± SD for triplicate cultures and are representative of three independent experiments. *P ≤ 0.05, **P ≤ 0.01, ***P ≤ 0.001, as compared with control.

We next examined pro‐inflammatory cytokine and chemokine secretion in our purified microglia and astrocyte populations exposed to cytosolic nucleic acids. Microglia produced robust CCL5 and IL‐6 in response to all 3 ligands (Fig. 5A,B), whereas only minimal levels of IL‐6 were produced by astrocytes in response to the Vv70mer (Fig. 5F). This may also account for the low levels of IL‐6 produced in response to this ligand in mixed glia which were comprised of ∼75% astrocytes however poly (dA:dT) and poly (I:C) produced significant levels of this cytokine. Microglia, but not astrocytes, produced TNF‐α and CCL3 and production of these cytokines was higher following transfection with the Vv70mer compared with either poly (dA:dT) and poly (I:C) (Fig. 5C,D). Finally, CXCL2 was secreted by astrocytes and not microglia (Fig. 5G), indicating the importance of astrocytes in recruiting microglia and other immune cells to the site of insult via the production of chemokines.

### Gene Silencing of cGAS and p204 Significantly Impairs IFN‐β Production in Primary Astrocytes and Microglia

**Figure 6:**
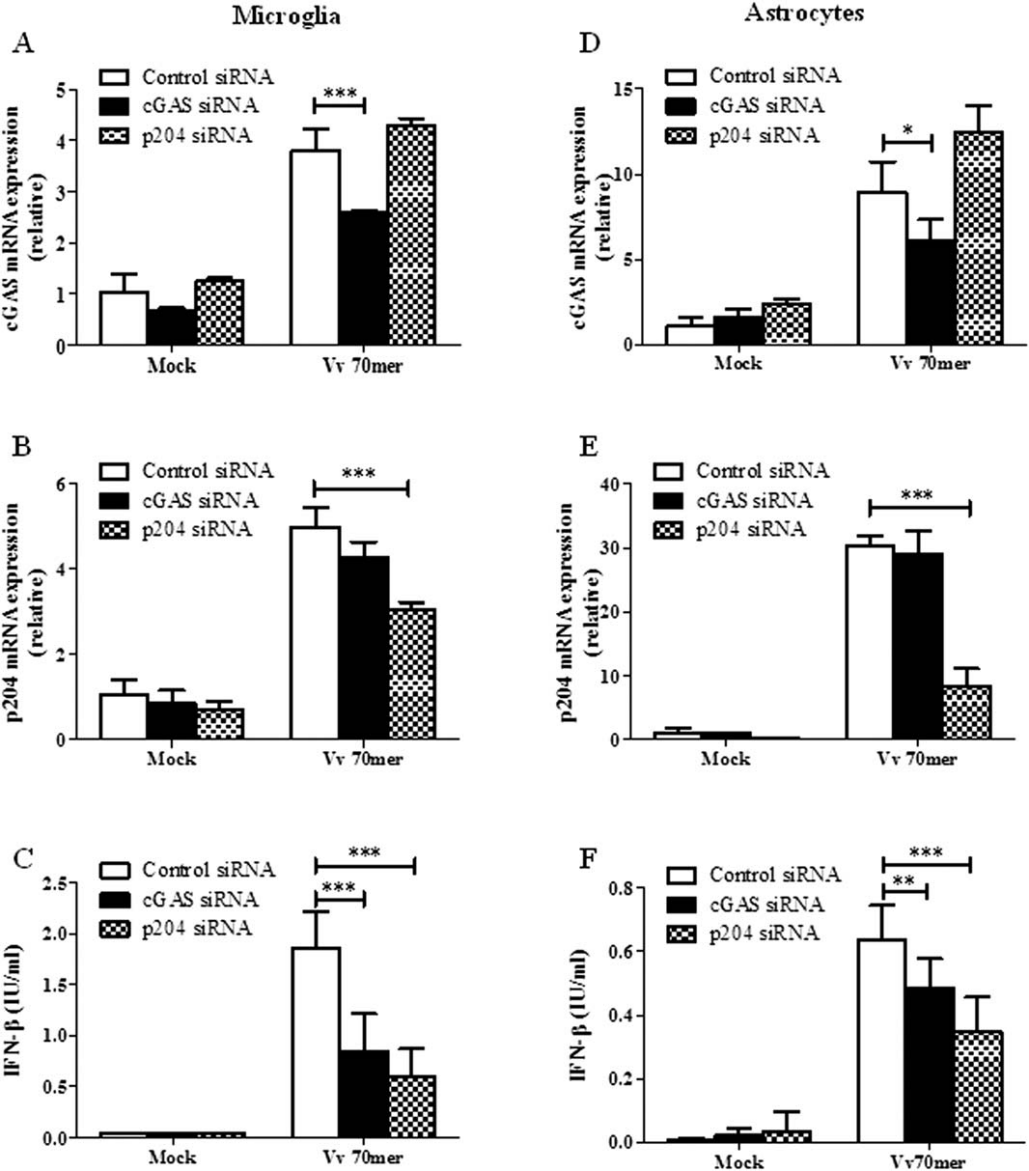
siRNA knockdown of cGAS and p204 significantly impairs IFN‐β production in primary astrocytes and microglia. Primary murine microglia (left panel) and astrocytes (right panel) were treated with siRNA against cGAS or p204 and then transfected with Vv70mer (1 μg/mL) for 6 h. Expression of cGAS (**A**, **D**) and p204 (**B**, **E**) was analyzed by quantitative PCR. Supernatants were harvested and IFN‐β production by microglia (C) and astrocytes (**F**) was quantified by ELISA. Results shown are means ± SD for triplicate cultures and are representative of three independent experiments. *P ≤ 0.05, **P ≤ 0.01, ***P ≤ 0.001, as compared with control siRNA.

Previous studies have confirmed that cGAS and p204 drive IFN‐β production in macrophages (Sun et al., 2013; Unterholzner et al., 2010). We carried out siRNA gene silencing in order to confirm a role for these sensors in driving interferon production in CNS immune cells. Vv70mer transfection induced robust expression of both cGAS and p204 in control siRNA treated microglia (Fig. 6A,B) and astrocytes (Fig. 6D,E) demonstrating that DNA can induce the expression of the sensors themselves as well as IFN‐β. Significant knockdown of cGAS and p204 was observed at 72 h (microglia) and 48 h (astrocytes) post siRNA transfection and while knockdown was incomplete, a significant reduction in IFN‐β expression was observed in both cases in response to DNA transfection (Fig. 6C,F). While it is likely that there is a certain level of redundancy between DNA sensors, this data demonstrates a direct involvement of these receptors in the response to immune stimulatory DNA.

### Nucleic Acid Driven IL‐6 is Partially Dependent on Activation of IFNAR in Primary Astrocytes

**Figure 7:**
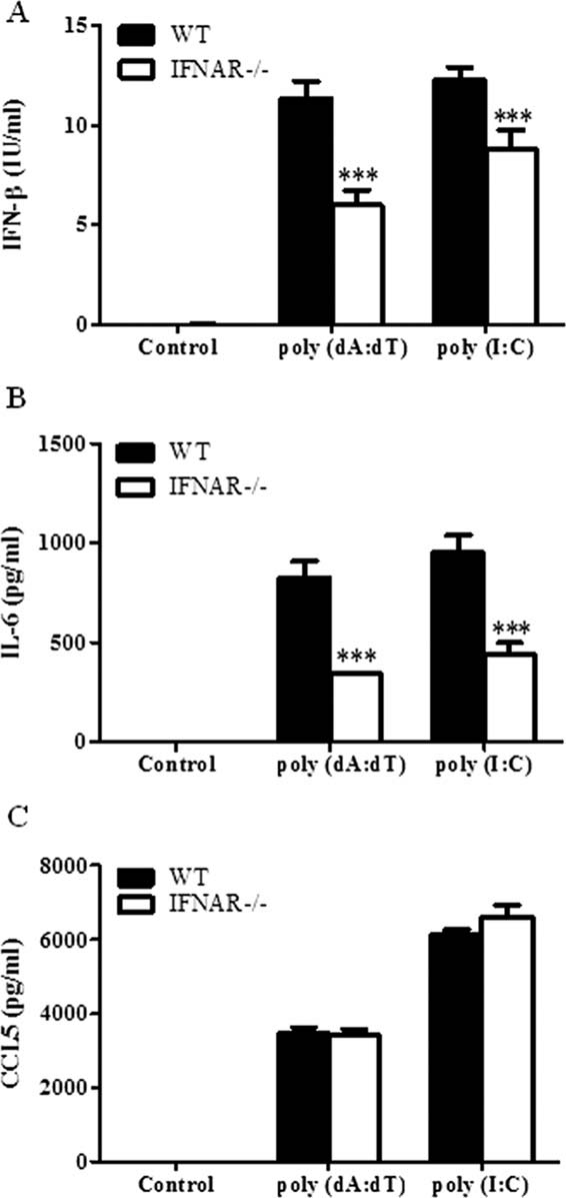
Nucleic acid induced IL‐6 production is dependent on activation of IFNAR in primary murine astrocytes. Primary murine astrocytes derived from wild type or IFNAR^−/−^ mice were transfected with poly (dA:dT) (1 μg/mL) or poly (I:C) (5 μg/mL) for 24 h. Supernatants were harvested and IFN‐β (**A**), IL‐6 (**B**), and CCL5 (**C**) production was quantified by ELISA. Results shown are means ± SD for triplicate cultures. ***P ≤ 0.001 as compared with control.

We had previously observed that IFN‐β is produced at 6 h following nucleic acid transfection in mixed glial cultures whereas IL‐6 production was not observed until after 24 h in response to (dA:dT) and poly (I:C) (Fig. 1A,B). We therefore sought to determine if production of IL‐6 is dependent on activation of the Type I IFN receptor. To examine this hypothesis, astrocytes were derived from both wild‐type and IFNAR deficient mice and transfected with poly (dA:dT) and poly (I:C) for 24 h. The levels of both IFN‐β and IL‐6 were significantly reduced in IFNAR deficient astrocytes as compared with their wild‐type counterparts suggesting that IL‐6 production is at least partially dependent on activation of the IFNAR. Furthermore, IFN‐β production itself is partially dependent on the IFN receptor confirming the presence of a positive feedback loop (Fig. 7A,B). In contrast, the production of the chemokine CCL5 does not appear to be dependent on Type I interferons (Fig. 7C).

### DNA Sensors are Upregulated In Vivo in an IFNAR Dependent Manner and by Neurodegeneration

**Figure 8:**
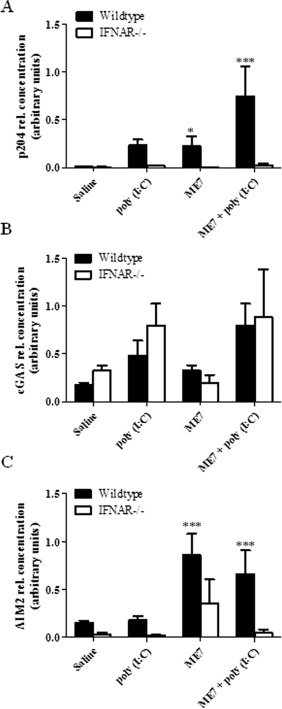
DNA sensors are up regulated *in vivo* in an IFNAR dependent manner and in the ME7 model of neurodegeneration. RNA was extracted from the perfused brains of control mice, poly (I:C) injected mice and ME7 mice (wild‐type, black bar; IFNAR^−/−^, white bar). P204 (**A**), cGAS (**B**), and AIM2 (**C**) mRNA levels were analyzed by quantitative PCR. Results shown are means ± SD. *P ≤ 0.05, ***P ≤ 0.001, for wild‐type compared with IFNAR^−/−^ mice.

We next examined the expression of the key antiviral DNA sensors (i.e., those sensors with functional human equivalents) *in vivo* following the peripheral administration of poly (I:C) as this is known to drive strong CNS IFN production in mice (Cunningham et al., 2007). RNA was extracted from perfused brain tissue from wild‐type and IFNAR^−/−^ mice 4 h after i.p. administration of poly (I:C) (12 mg/kg) and quantitative PCR was performed. Both p204 and cGAS were upregulated in wild‐type mice challenged with poly (I:C) as compared with control mice (Fig. 8A,B). A minor increase in AIM2 expression was also observed however this was not significant (Fig. 8C). p204 expression in response to poly (I:C) was dependent on activation of the IFNAR, as expression was significantly lower in IFNAR deficient mice (Fig. 8A).

It has been postulated that DNA can exacerbate neurodegenerative conditions such as Alzheimer's and Parkinson's disease (Heneka et al., 2014). We have previously demonstrated that Type I IFNs are elevated and active in the ME7 prion model of chronic neurodegeneration (Field et al., 2010). This model involves injection of 10% (w/v) ME7‐infected C57BL/6 brain homogenate to the dorsal hippocampus of mice after which neuronal loss, astrogliosis, and microglial activation peak in the hippocampus and posterior thalamus at ∼18 weeks (Asuni et al., 2014; Cunningham et al., 2005). RNA was extracted from perfused brain tissue from wild‐type and IFNAR^−/−^ mice, with and without ME7 prion disease, and quantitative PCR was performed. Expression of p204 and AIM2 was upregulated in an IFNAR dependent manner in the ME7 mice (Fig. 8A,C); however, expression of cGAS was similar between wild‐type and IFNAR^−/−^ mice (Fig. 8B). Mice were administered poly (I:C) systemically in order to mimic systemic viral infection during a chronic neurodegenerative condition since this has been shown to exacerbate disease (Field et al., 2010). poly (I:C) administration enhanced p204 and AIM2 expression in ME7 mice. This increase was also dependent on activation of IFNAR (Fig. 8A,C). The expression of cGAS did increase in ME7 mice with poly (I:C); however, this does not appear to be dependent on activation of the interferon receptor. Interestingly, the constitutive expression of AIM2 is lower in IFNAR^−/−^ mice as compared with wild‐type mice. This may be a result of low basal levels of Type I IFNs inducing expression of this sensor in the CNS.

## Discussion

We have found that the PYHIN family of proteins and cGAS are expressed in both astrocytes and microglia at the mRNA level and that many of these sensors are highly upregulated in response to IFN‐β treatment. Exposure of these cells to immune stimulatory nucleic acids results in robust production of pro‐inflammatory mediators and antiviral cytokines. Astrocytes are particularly responsive to double stranded RNA suggesting that this cell type plays a vital role in the recognition of double stranded RNA viruses. Furthermore, IL‐6 production by astrocytes in response to both poly (dA:dT) and RNA is likely to be a secondary event following the production of IFN‐β and activation of the IFNAR. Previous studies have demonstrated a protective role for IL‐6 during HSV‐1 infection (Chucair‐Elliott et al., 2014).We have also observed a strong induction of the chemokine, CCL5, which as well as playing a vital role in antiviral responses has also been shown to mediate cerebral inflammation and tissue injury following focal ischemia‐reperfusion (Denes et al., 2010; Terao et al., 2008). Interestingly, CCL5 induction following DNA stimulation was not dependent on IFN‐β as no difference was seen in CCL5 production between wild‐type and IFNAR deficient astrocytes. DNA transfection also led to an increase in the expression of the key sensors p204 and cGAS in both astrocytes and microglia and given the strong induction of IFN‐β by DNA, this is also likely to be dependent on IFNAR.

It has previously been demonstrated that microglia and IFN‐β are required to counter HSV‐1‐driven brain lateral ventricle enlargement and encephalitis (Conrady et al., 2013). Furthermore, mice lacking cGAS have increased mortality and viral titres in the brain following HSV‐1 infection (Li et al., 2013) thus supporting a role for IFN‐β and nucleic acid sensors in CNS infection control. In contrast, nucleic acid sensors have been implicated in the recognition of host‐derived nucleic acids that are aberrantly localized or released under conditions of cellular stress or injury and are therefore thought to negatively impact on a number of disease pathologies (de Rivero Vaccari et al., 2012; Heneka et al., 2014). Given the susceptibility of neurons to cell death on exposure to pro‐inflammatory mediators, the recognition of host derived DNA in addition to various self molecules that are present in degenerating brains has implications for chronic neurodegenerative conditions such as Alzheimer's and Parkinson's disease as well as more acute conditions such as stroke and traumatic brain injury. Indeed it has been demonstrated that cell free DNA is increased in the CSF of patients following TBI and that application of this fluid to cortical neurons results in AIM2 activation (Adamczak et al., 2014). Additionally, an increase in cell‐free DNA and IL‐6 has been observed in patients with focal epilepsy (Liimatainen et al., 2013) while chronic over production of IL‐6 is a characteristic of temporal lobe epilepsy (Liimatainen et al., 2009). We have assessed the basal expression of p204, AIM2, and cGAS in normal brain tissue and in mice challenged systemically with poly (I:C) to drive IFN‐β production. All three sensors are expressed at the basal level and expression is enhanced following poly (I:C) challenge. Furthermore, in line with our *in vitro* data, gene expression is dependent on Type I interferon signalling. Finally, we have examined the expression of the key DNA sensors in the ME7 model of prion disease, which displays robust micro‐ and astrogliosis as well as significant chronic neurodegeneration (Asuni et al., 2014; Cunningham et al., 2005; Field et al., 2010). Both AIM2 and p204 were significantly upregulated in diseased brains in the regions where astrogliosis and microgliosis is evident and expression was enhanced further in those mice administered with poly (I:C). It will be of interest to examine whether both astrocytes and microglia express DNA sensors in this model. There are currently limitations to performing coimmunohistochemical staining due to a lack of commercially available antibodies; however, these results indicate a potential for heightened responses to host derived products, which could not only potentiate underlying inflammation but exacerbate existing neuroinflammation. It is striking that in the ME7 model of prion disease, systemic poly I:C induces exaggerated CNS Type I IFN responses and IL‐1 production (Field et al., 2010), consistent with the idea that during disease nucleic acid sensors are upregulated and subsequent inflammatory stimulation can drive excessive production of these mediators. Given the robust expression of AIM2 both *in vitro* and *in vivo*, it will be of interest to examine DNA induced IL‐1 production in this and other models of CNS pathology. It remains to be seen if these sensors are elevated in other models of neurodegeneration and in human patient samples but it is important to state that predictions arising from the ME7 model, about the impact of systemic inflammation on progression of neurodegeneration (Cunningham et al., 2009; Field et al., 2010) have been borne out in the human Alzheimer's disease population (Holmes et al., 2009).

In conclusion, while the recognition of nucleic acids plays a vital role in the defence against CNS pathogens, triggering of innate immune mechanisms is now emerging as a crucial component of major neurodegenerative conditions (Field et al., 2010; Khorooshi and Owens, 2010; Wang et al., 2011). Microglia and astrocytes express the machinery required to respond to DNA and RNA; however, this is nondiscriminate. Therefore, in the absence of infection, the use of anti‐inflammatory therapies may be a useful treatment approach for neurodegenerative diseases where innate immune activation may be an early cause rather than a late consequence of the condition. In the case of CNS infection, while recognition of microbial PAMPs in an appropriate immune response, the sustained activation of microglia and production of pro‐inflammatory mediators can impact directly on neighboring neurons leading to cell death, suppressed axonal transport and impaired neurogenesis. Understanding the precise contribution of PRRs to pathogen sensing in the brain will contribute to the development of tailor made treatments with optimal antiviral as well as anti‐inflammatory properties.
